# Malnutrition and Elevated Mortality Among Refugees from South Sudan — Ethiopia, June–July 2014

**Published:** 2014-08-15

**Authors:** Ellen Andresen, Oleg O. Bilukha, Zeray Menkir, Megan Gayford, Millicent Kavosa, Mulugeta Wtsadik, Gidraf Maina, Mesfin Gose, Irene Nyagucha, Cyrus Shahpar

**Affiliations:** 1 United Nations High Commissioner for Refugees; 2 Division of Global Health Protection, Center for Global Health, CDC; 3 Administration for Refugee and Returnee Affairs, Ethiopia; 4 World Food Programme; 5 United Nations Children’s Fund

As a result of armed civil conflict in South Sudan that started in mid-December of 2013, an estimated 1.1 million persons were internally displaced, and approximately 400,000 refugees fled South Sudan to neighboring countries (primarily to Ethiopia, Uganda, Sudan, and Kenya). Refugees from South Sudan arriving in Ethiopia are sheltered in three refugee camps located in Gambella region: Leitchuor, Kule, and Tierkidi. The camps were established during January–May 2014 and have estimated refugee populations of 47,000, 51,000, and 50,000, respectively. Reports from health clinics and humanitarian agencies providing assistance to refugees suggested poor nutritional status of arriving refugees and elevated mortality rates. To assess the nutritional status of refugee children aged 6–59 months and mortality rates (crude [all ages] and aged <5 years), the Administration for Refugee and Returnee Affairs (an Ethiopian government aid agency), the United Nations High Commissioner for Refugees, World Food Programme, and United Nations Children’s Fund, in collaboration with CDC, conducted cross-sectional population-representative surveys in Leitchuor, Kule, and Tierkidi camps during June–July 2014. Anthropometric measurements in children were taken using standard procedures (1), and nutritional status was classified based on 2006 World Health Organization (WHO) growth standards (2). Hemoglobin was measured using HemoCue Hb 301 (3). Anemia was diagnosed according to WHO thresholds (4). Retrospective mortality rates in Leitchuor and Kule were measured using a household census method.

Prevalence of global acute malnutrition among children aged 6–59 months ranged from 25.8% in Leitchuor to 30.3% in Kule, approximately twice the WHO emergency threshold of 15% (5). Prevalence of severe acute malnutrition also was very high, ranging from 5.7% in Leitchuor to 10.0% in Kule (Table). Crude (all ages) and aged <5 years mortality rates substantially exceeded emergency thresholds of 1 and 2 per 10,000 per day, respectively (6), in both Leitchuor and Kule (Table). Anemia prevalence among children aged 6–59 months in all camps exceeded 40%, indicating a problem of high public health significance according to WHO classification (4) (Table).

These survey results indicate a serious public health emergency among refugees from South Sudan residing in the three camps in Ethiopia. In response to the large influx of refugees into Ethiopia, the Administration for Refugee and Returnee Affairs, the United Nations High Commissioner for Refugees, and other humanitarian agencies established essential health services and nutrition treatment programs coupled with active screening for malnutrition. Blanket supplementary feeding programs targeting young children and pregnant and lactating women were established in all camps. Efforts directed at strengthening outreach activities to detect malnourished children, decentralizing health and nutrition services to improve access, and increasing awareness of the refugee population regarding available blanket feeding programs will be implemented with the goal to improve health and nutrition outcomes and decrease mortality. All registered refugees in the camps are receiving food aid assistance from the World Food Programme, and the planned decentralization of distributions as well as family-targeted distributions (as opposed to group distributions) will aim to improve the overall food security of vulnerable families.

## Figures and Tables

**TABLE:** Mortality rates and prevalence of global acute malnutrition and anemia in child refugees aged 6–59 months from South Sudan — three refugee camps, Ethiopia, June 2014

	Leitchuor camp	Kule camp	Tierkidi camp
			
Nutrition standard	% (95% CI)	% (95% CI)	% (95% CI)
**Global acute malnutrition, children aged 6–59 mos** *			
Total (WHZ <-2 or bilateral pitting edema)	25.8 (21.5–30.6)	30.3 (25.8–35.2)	28.0 (23.9–32.5)
Moderate (WHZ -3 to <-2)	20.1 (16.3–24.6)	20.3 (16.4–24.7)	20.2 (16.6–24.3)
Severe (WHZ<-3 or bilateral pitting edema)	5.7 (3.7–8.6)	10.0 (7.3–13.5)	7.8 (5.6–10.8)
**Anemia, children aged 6–59 mos** †			
Any anemia (Hb <11.0 g/dl)	42.7 (37.8–47.7)	51.9 (46.8–57.0)	46.2 (41.5–51.1)
Mild (Hb 10 to <11.0 g/dl)	22.4 (18.4–27.0)	28.0 (23.6–32.8)	26.6 (22.6–31.1)
Moderate (Hb 7 to <10.0 g/dl)	19.9 (16.1–24.4)	23.4 (19.3–28.0)	19.1 (15.3–23.2)
Severe (Hb <7.0 g/dl)	0.3 (0.0–1.6)	0.5 (0.1–2.0)	0.5 (0.1–1.7)
**Mortality**			
Crude mortality rate§,¶	1.54 (0.99–2.40)	1.63 (1.08–2.46)	—
Aged <5 yrs mortality rate**,††	4.07 (2.28–7.19)	5.64 (3.49–9.03)	—

**Abbreviations:** CI = confidence interval; WHZ = weight-for height z-score; Hb = hemoglobin.

* Sample sizes: Leitchuor, 353; Kule, 360; Tierkidi, 411.

† Sample sizes: Leitchuor 361, Kule, 368; Tierkidi, 413.

§ Deaths per 10,000 persons per day.

¶ Sample sizes: Leitchuor, 2,060; Kule, 2,078.

** Deaths per 10,000 children aged <5 years per day.

†† Sample sizes: Leitchuor, 460; Kule, 446.
